# Early and Long-Term Outcomes of Patients Undergoing Surgery for Native and Prosthetic Valve Endocarditis: The Role of Preoperative Neutrophil-to-Lymphocyte Ratio, Neutrophil-to-Platelet Ratio, and Monocyte-to Lymphocyte Ratio

**DOI:** 10.3390/jcm14020533

**Published:** 2025-01-16

**Authors:** Antonella Galeone, Jacopo Gardellini, Venanzio Di Nicola, Fabiola Perrone, Maria Serena Menzione, Renato Di Gaetano, Giovanni Battista Luciani

**Affiliations:** 1 Department of Surgery, Dentistry, Pediatrics and Gynecology, Division of Cardiac Surgery, University of Verona, 37126 Verona, Italy; 2 Department of Cardiology, Azienda Sanitaria dell’Alto Adige, 39100 Bolzano, Italy

**Keywords:** infective endocarditis, neutrophil-to-lymphocyte ratio, surgical outcome

## Abstract

**Background/Objectives:** Previous studies evaluated the prognostic role of hematological parameters in predicting outcome in patients with infective endocarditis (IE). However, only a few studies evaluated the role of hematological parameters in patients undergoing surgery for IE. The aim of this study was to review our 20-year experience with the treatment of native (NVE) and prosthetic (PVE) valve endocarditis and to evaluate the role of neutrophil-to-lymphocyte ratio (NLR), neutrophil-to-platelet ratio (NPR), platelet-to-lymphocyte ratio (PLR), monocyte-to-lymphocyte ratio (MLR), neutrophil-to monocyte ratio (NMR), and systemic inflammatory index (SII) on early and long-term outcomes of patients undergoing surgery for NVE and PVE. **Methods:** All adult patients undergoing surgery for NVE and PVE at our institution between January 2001 and December 2022 were included in the study. Preoperative NLR, NPR, PLR, MLR, NMR, and SII were calculated using hemograms with complete blood count. **Results:** During the study period, 503 patients, 371 (74%) males, median age 65 (52–73) years, underwent surgery for NVE (n = 337, 67%) or PVE (n = 166, 33%). Patients with PVE had significantly more aortic annulus abscess (95 (57%) vs. 51 (15%); *p* < 0.001), longer CPB (180 (131–235) vs. 105 (84–145) min; *p* < 0.001) and aortic cross-clamping times (129 (96–175) vs. 82 (64–114) min; *p* < 0.001), and received more aortic homografts (47 (28%) vs. 28 (9%); *p* < 0.001) and postoperative pacemaker implantation (25 (15%) vs. 20 (6%); *p* < 0.001 compared to patients with NVE. Preoperative NLR was 3.7 (2.4–6.1), NPR was 23 (16–37), PLR was 159 (106–210), NMR was 8.4 (6.6–12), MRL was 0.41 (0.29–0.62], and SII was 790 (485–1396). NLR, NPR, and MLR were significantly lower in patients with NVE compared to patients with PVE and in survivors compared to non-survivors. Overall mean survival time was 12.2 ± 0.5 years, with patients with NVE having better early and late survival compared to patients with PVE. Patients with preoperative NLR < 3.8, NPR < 30.9, and MLR < 0.4 had significantly better mean survival time compared to patients with preoperative NLR > 3.8, NPR > 30.9, and MLR > 0.4, respectively. **Conclusions:** In patients undergoing surgery for IE, preoperative higher NLR, NPR, and MLR are associated with increased early and long-term mortality.

## 1. Introduction

Infective endocarditis (IE) represents a challenging condition with an increasing incidence that reached 13.8 cases per 100,000 subjects per year in 2019 [1]. Despite diagnostic and therapeutic advances, IE is associated with high early and long-term mortality and morbidity, with 14–22% in-hospital mortality rates and up to 50% mortality at 10 years [2]. Surgery is required in 30–50% of the patients affected by IE, and surgical mortality ranges from 15% to 45%. Patients with prosthetic valve endocarditis (PVE) have an in-hospital mortality rate that is twice as high with more postoperative complications as compared with patients with native valve endocarditis (NVE) [3,4,5]. Early risk stratification is of paramount importance in patients with IE to determine optimal timing of surgery and to reduce complications and mortality rates. The potential diagnostic and prognostic roles of several biomarkers have been evaluated, including, among others, C-reactive protein, procalcitonin levels, interleukin (IL)-6, natriuretic brain peptides, and troponins [6,7], which reflect the complex pathophysiology of the disease involving not only inflammatory responses but also circulatory and end-organ dysfunction. The prognostic role of hematological parameters in predicting outcome in patients with IE has also been investigated [8,9,10]. However, only a few studies evaluated the role of hematological parameters in patients undergoing surgery for IE. Therefore, the aim of this study was to review our 20-year experience with the treatment of NVE and PVE and to evaluate the role of neutrophil-to-lymphocyte ratio (NLR), neutrophil-to-platelet ratio (NPR), platelet-to-lymphocyte ratio (PLR), monocyte-to-lymphocyte ratio (MLR), neutrophil-to monocyte ratio (NMR), and systemic inflammatory index (SII) on early and long-term outcomes of patients undergoing surgery for IE.

## 2. Materials and Methods

The study was conducted in accordance with the Declaration of Helsinki and approved by the Ethics Committee of the Azienda Ospedaliera Universitaria Integrata of Verona (approval number: 64927; approval date: 30 November 2020).

All consecutive, adult patients undergoing surgery for native (NVE) and prosthetic valve endocarditis (PVE) at our institution between January 2001 and December 2022 were included in the study. Patients’ characteristics, perioperative data, and in-hospital outcomes were extracted from patients’ paper-based and electronic medical records. The diagnosis of NVE and PVE was based on the revised Duke’s criteria [11]. Active IE was defined as an IE requiring iv antibiotics. Early PVE was defined as an IE occurring within 1 year after the prior surgery. Indications for surgery included heart failure, uncontrolled infection, and prevention of embolism according to the most recent available international recommendations [1].

We performed all operations through a median full sternotomy, using standard cardiopulmonary bypass (CPB) and cold blood or crystalloid cardioplegia. Native valve endocarditis was managed by valve replacement or repair according to intraoperative findings and surgeon’s choice. Prosthetic valve endocarditis was managed by valve replacement with biological or mechanical prosthesis. In case of aortic PVE complicated by extensive aortic root abscess requiring aortic root replacement (ARR), fistulae, and/or multiple valve involvement, prosthetic valve conduit or cryopreserved aortic homografts (CAH) were used according to anatomical findings and surgeon’s preference. Aortic homografts were implanted using the full-root, the free-hand sub-coronary with intact non-coronary sinus, or the intraluminal cylinder technique. In the presence of discontinuity of the mitro-aortic curtain and fistulae, we used a bovine pericardial patch or the anterior mitral leaflet of the CAH to repair the defect. In case of left ventricle to right atrium or to the right ventricular outflow tract fistulae, we performed an extensive debridement through both the aortic root and the right atrium or the infundibulum, as requested. The residual defect was closed with a pericardial bovine patch on the right side and with the anterior mitral leaflet of the CAH on the left side. All the CAH were provided by the Treviso Tissue Bank Foundation (Treviso, Italy).

In patients with a preoperative hemogram with complete blood count, the hematological parameters were calculated according to the following formulas: NLR (absolute neutrophils count (×10^9^/L)/absolute lymphocytes count (×10^9^/L)), NPR (neutrophil count (×10^9^/L) × 1000/platelet count (×10^9^/L)), PLR (platelet count (×10^9^/L)/lymphocyte count (×10^9^/L)), NMR (neutrophil count (×10^9^/L/monocyte count (×10^9^/L)), MLR (monocyte count (×10^9^/L)/lymphocyte count (×10^9^/L)), and SII (platelet count (×10^9^/L) × neutrophil count (×10^9^/L)/lymphocyte count (×10^9^/L)) were calculated.

Follow-up data, including routine visits and subsequent hospitalization, were collected until December 2023 from cardiology reports and hospital records or via phone and e-mail contact with patients, family members, family physicians, and cardiologists. The follow-up time was calculated either to death or to the last verified contact with the patient. Clinical outcomes of interest included mortality and reintervention for bioprosthetic valve dysfunction (BVD) or other causes. Mortality was defined as periprocedural (occurring ≤ 30 days after the index procedure or > 30 days but during the index hospitalization), early (occurring > 30 days but ≤ 1 year after the index hospitalization), and late mortality (occurring > 1 year after the index hospitalization), according to Valve Academic Research Consortium 3 (VARC-3) [12]. BVD was defined as the presence of structural valve dysfunction (SVD), non SVD (NSVD), infective endocarditis, and thrombosis [12].

### Statistical Analysis

Categorical variables are expressed as numbers and percentages and compared with χ^2^ test. Continuous variables with a skewed distribution are presented as median and interquartile range and compared with Mann-Whitney U test.

The receiver operating characteristic (ROC) analysis was used to assess the ability of the hematological parameters in predicting mortality. The area under the ROC (AUC) curve and corresponding 95% confidence interval (CI) were calculated. The optimal cutoff values were determined with the Youden Index, and their sensitivity and specificity were calculated.

The Kaplan–Meier method was used to draw survival curves; the log-rank test was used to compare survival among groups. The Reverse Kaplan–Meier survival curve was used to calculate follow-up rate. Hazard ratios for mortality were determined by univariate and multivariate Cox proportional hazards regression analysis with data presented as hazard ratio with 95%. A two-tailed *p* value < 0.05 was taken to indicate statistical significance. Completeness of the follow-up was measured according to Clark’s formula [13]. Statistical analysis was performed using Sigmaplot version 14.0 (Systat Software Inc., San Jose, CA, USA).

## 3. Results

### 3.1. Demography

Five hundred three patients, 74% males, median age 65 (52–73) years, underwent surgery for NVE (n = 337, 63%) or PVE (n = 166, 33%) at our institution during the study period; 165 (33%) patients (108 (65%) NVE and 57 (35%) PVE) were operated from 2001 to 2011, and 338 (67%) patients (229 (68%) NVE and 109 (32%) PVE) were operated from 2012 to 2022. Isolated microorganisms from blood and/or native or prosthetic valve cultures are listed in Table 1. Streptococci were the most common cause of IE in patients with NVE, while coagulase negative staphylococci were the most common cause of IE in patients with PVE.

Pre, intra, and perioperative patients’ characteristics are illustrated in Table 2. Patients with PVE were significantly older than patients with NVE (70 (63–75) vs. 61 (49–71) years; *p* < 0.001). The aortic valve was the most common affected valve in patients with IE (n = 352, 70%), and patients with PVE had significantly more aortic valve IE compared to patients with NVE (85% vs. 62%, respectively; *p* < 0.001). Conversely, the mitral valve was more affected in patients with NVE compared to patients with PVE (51% vs. 25%, respectively; *p* < 0.001). Patients with PVE had significantly more aortic annulus abscess (95 (57%) vs. 51 (15%); *p* < 0.001), longer CPB (180 (131–235) vs. 105 (84–145) min; *p* < 0.001), and aortic cross-clamping times (129 (96–175) vs. 82 (64–114) min; *p* < 0.001) and received more aortic homografts (47 (28%) vs. 28 (9%); *p* < 0.001) and postoperative pacemaker implantation (25 (15%) vs. 20 (6%); *p* < 0.001) compared to patients with NVE (Table 2).

### 3.2. Hematological Parameters

Preoperative hemograms with complete blood counts were available in 317 (63%) patients. Preoperative NLR, NPR, PLR, NMR, MLR, and SII were calculated, and median values are illustrated in Table 3. No difference was found in hematological parameters between 53 patients with preoperative negative blood culture and 264 patients with preoperative positive blood culture except for NMR (NLR 3.5 vs. 3.7, *p* = 0.3; NPR 22 vs. 24, *p* = 0.5; PLR 155 vs. 161, *p* = 0.4; NMR 7.7 vs. 8.5, *p* = 0.02; MLR 0.42 vs. 0.41, *p* = 0.2; SII 695 vs. 809, *p* = 0.2). Patients with PVE had significantly higher values of NLR, NPR, and MLR compared to patients with NVE. Additionally, patients who died after surgery or during the follow-up had significantly higher preoperative values of NLR, NPR, and MLR compared to patients who survived after surgery and during the follow-up period.

**Figure 1:**
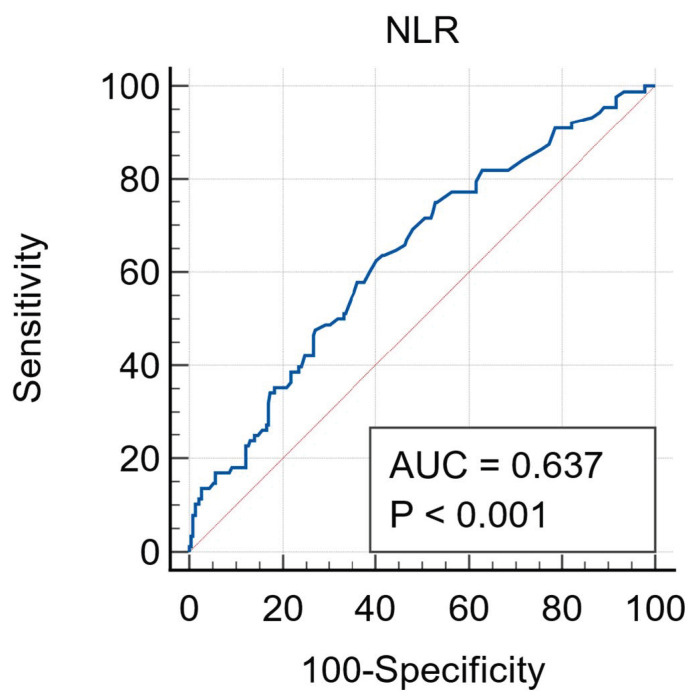
ROC curve analysis for NLR. ROC: receiver operating characteristics. NLR: neutrophil-lymphocyte ratio.

The median NLR was significantly lower in the survivors than in non-survivors (3.3 [2.3–5.5] vs. 4.6 [2.9–7.6]; *p* < 0.001). The ROC curve analysis showed that NLR higher than 3.8 had 62.5% sensitivity and 59.8% specificity in predicting mortality (AUC = 0.637, 95% CI: 0.58–0.69, *p* < 0.001) (Figure 1).

**Figure 2:**
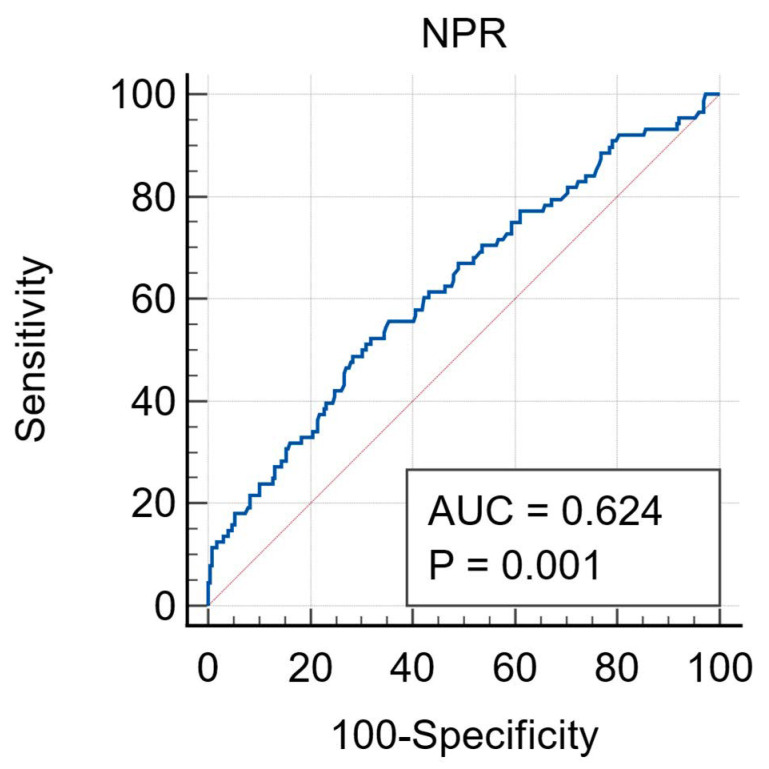
ROC curve analysis for NPR. ROC: receiver operating characteristics. NPR: neutrophil-platelet ratio.

The median NPR was significantly lower in the survivors than in non-survivors (22 (15–34) vs. 29 (18–49); *p* < 0.001). The receiver operating characteristics (ROC) curve analysis showed that NPR higher than 30.9 had 48.9% sensitivity and 71.6% specificity in predicting mortality (AUC = 0.624, 95% CI: 0.57–0.68, *p* < 0.001) (Figure 2).

**Figure 3:**
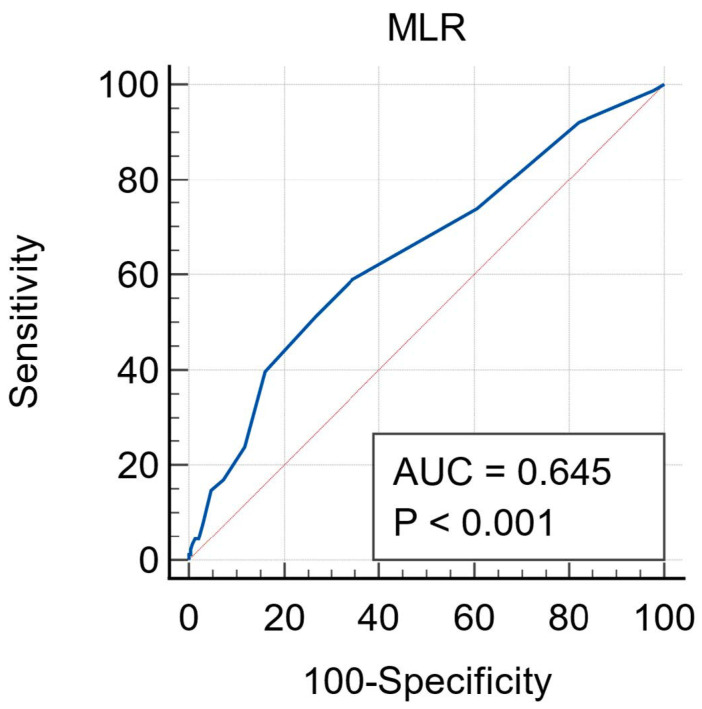
ROC curve analysis for MLR. ROC: receiver operating characteristics. MLR: monocyte-lymphocyte ratio.

The median MLR was significantly lower in the survivors than in non-survivors (0.39 (0.28–0.58) vs. 0.55 (0.33–0.75) *p* < 0.001). The receiver operating characteristics (ROC) curve analysis showed that NPR higher than 0.4 had 59.1% sensitivity and 65.5% specificity in predicting mortality (AUC = 0.645, 95% CI: 0.59–0.69, *p* < 0.001) (Figure 3).

No difference was observed between survivors and non survivors for PLR (AUC = 0.5; *p* = 0.9), NMR (AUC = 0.52; *p* = 0.5), and SII (AUC = 0.55; *p* = 0.17).

### 3.3. Survival

Two hundred eight (41%) patients died during the follow-up period, 128 (25%) patients with NVE and 80 (16%) patients with PVE. We recorded 50 (10%) periprocedural deaths, 32 (6%) early deaths, and 126 (25%) late deaths.

Three patients were lost during the follow-up period. The completeness of the follow-up was 98.8%, according to Clark’s formula.

**Figure 4:**
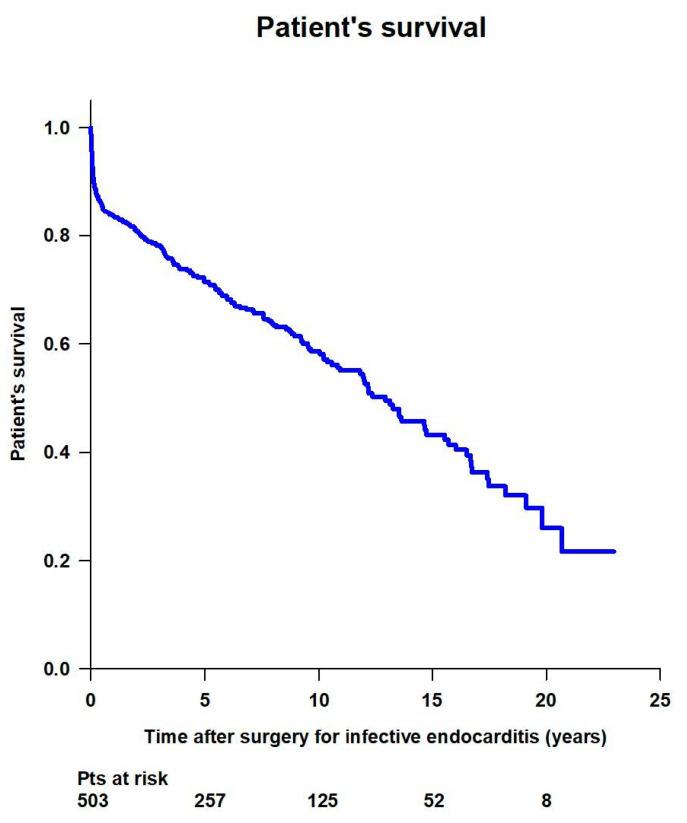
Patient’s survival after surgery for infective endocarditis.

Overall mean survival time was 12.2 ± 0.5 years, and survival rates were 92.8% at 30 days, 83.7% at 1 year, 71.4% at 5 years, 58.6% at 10 years, 43.2% at 15 years, and 26% at 20 years (Figure 4).

**Figure 5:**
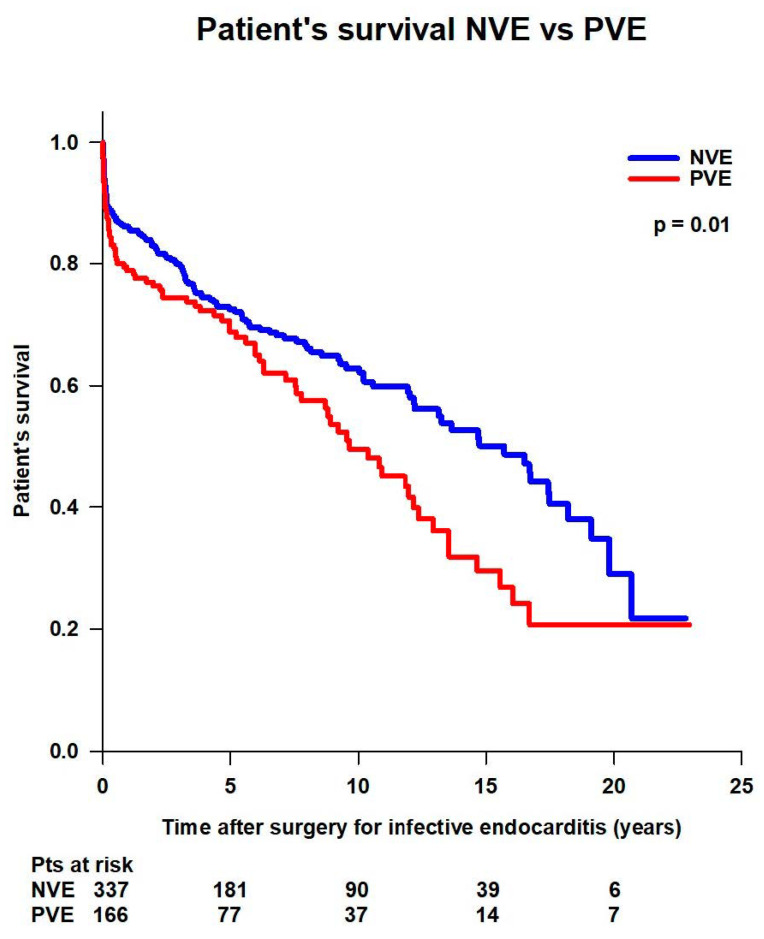
Patient’s survival after surgery for native (NVE) or prosthetic (PVE) valve endocarditis.

Patients with NVE had significantly better mean survival time (13 ± 0.6 years vs. 10.5 ± 0.8 years; *p* = 0.01) and survival rates (93.5% at 30 days, 86.1% at 1 year, 72.5% at 5 years, 62.9% at 10 years, and 50.1% at 15 years, and 34.8% at 20 years vs. 91.6% at 30 days, 78.8% at 1 year, 68.8% at 5 years, 49.6% at 10 years, and 29.6% at 15 years, and 20.7% at 20 years) compared to patients with PVE (Figure 5).

**Figure 6:**
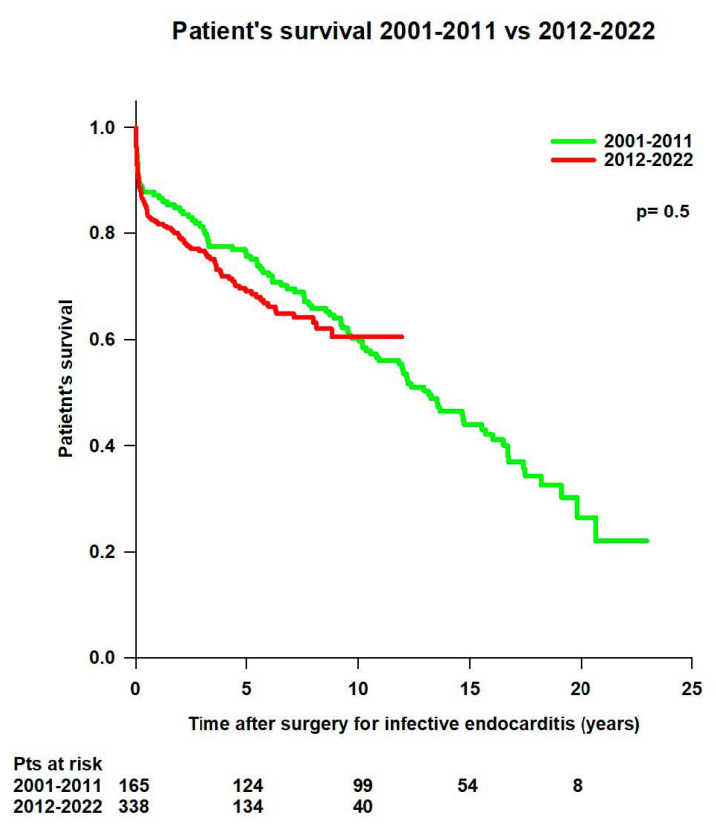
Patient’s survival according to the period of surgery for infective endocarditis (2001–2011 vs. 2012–2022).

No difference was observed between patients operated in the first period of the study (2001–2011, n = 165) and those operated in the second period of the study (2012–2022, n = 338) in mean survival time (12.6 ± 0.7 years vs. 8.3 ± 0.3 years; *p* = 0.5) and survival rates (94.5% at 30 days, 87.3% at 1 year, 76.3% at 5 years, 60.3% at 10 years, 44.8% at 15 years, and 26.4% at 20 years vs. 92% at 30 days, 81.9% at 1 year, 69% at 5 years, and 60.5% at 10 years) (Figure 6).

Univariate analysis was performed with pre and perioperative variables. Significant variables at univariate analysis were entered in the Cox multivariate regression. Multivariate analysis showed that age > 65 years, postoperative ECMO, and postoperative CRRT were independent predictors of mortality (Table 4).

**Figure 7:**
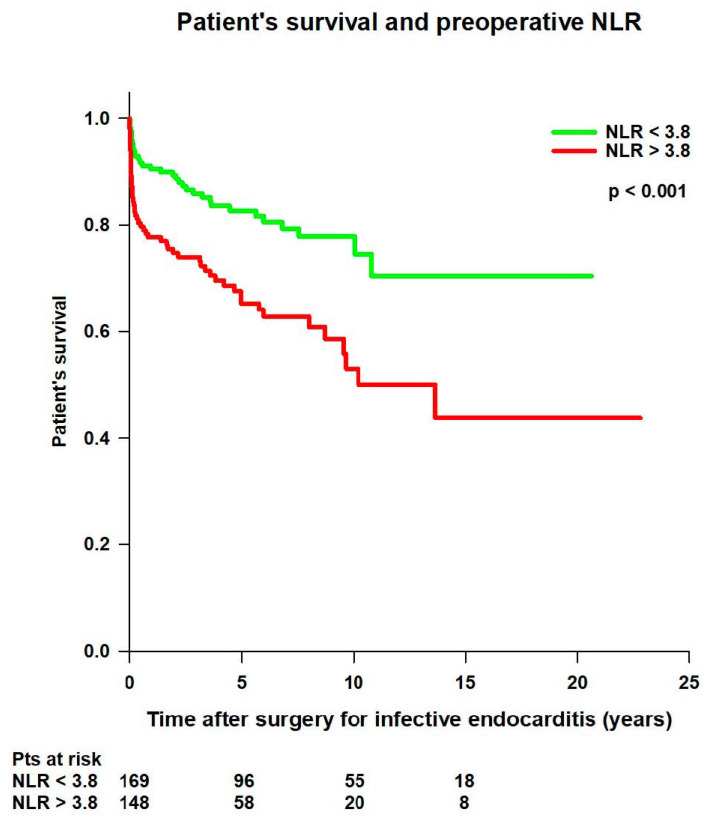
Patient’s survival after surgery for infective endocarditis, according to preoperative neutrophil-to-lymphocyte ratio (NLR) value.

Patients with preoperative NLR < 3.8 had significantly better mean survival time (15.9 ± 0.8 vs. 12.6 ± 1.1 years; *p* < 0.001) compared to patients with preoperative NLR > 3.8 (Figure 7).

**Figure 8:**
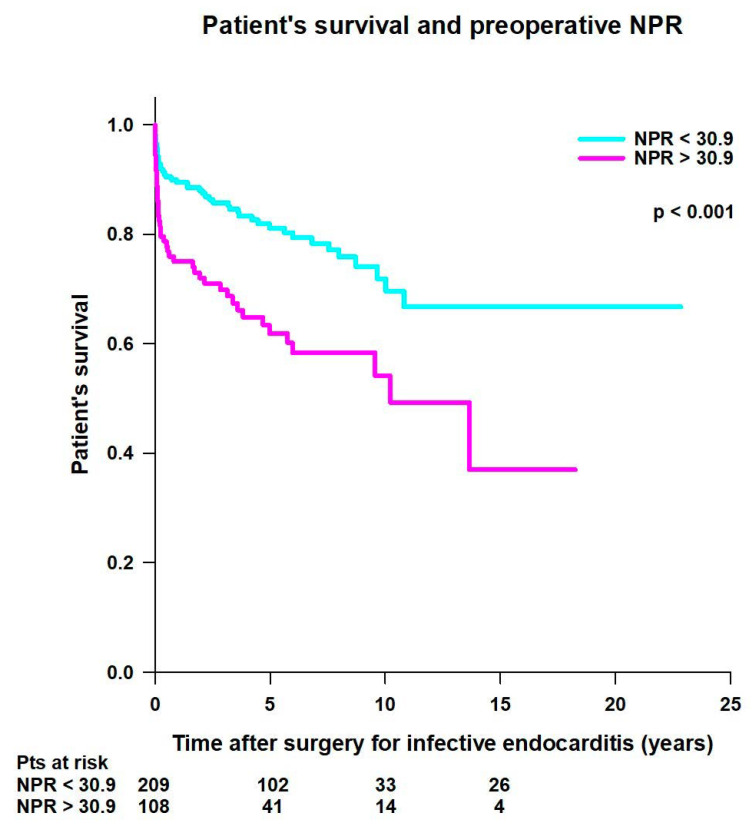
Patient’s survival after surgery for infective endocarditis, according to preoperative neutrophil-to-platelet ratio (NPR) value.

Patients with preoperative NPR < 30.9 had significantly better mean survival time (16.8 ± 0.8 years vs. 10 ± 1 years; *p* < 0.001) compared to patients with preoperative NPR > 30.9 (Figure 8).

**Figure 9:**
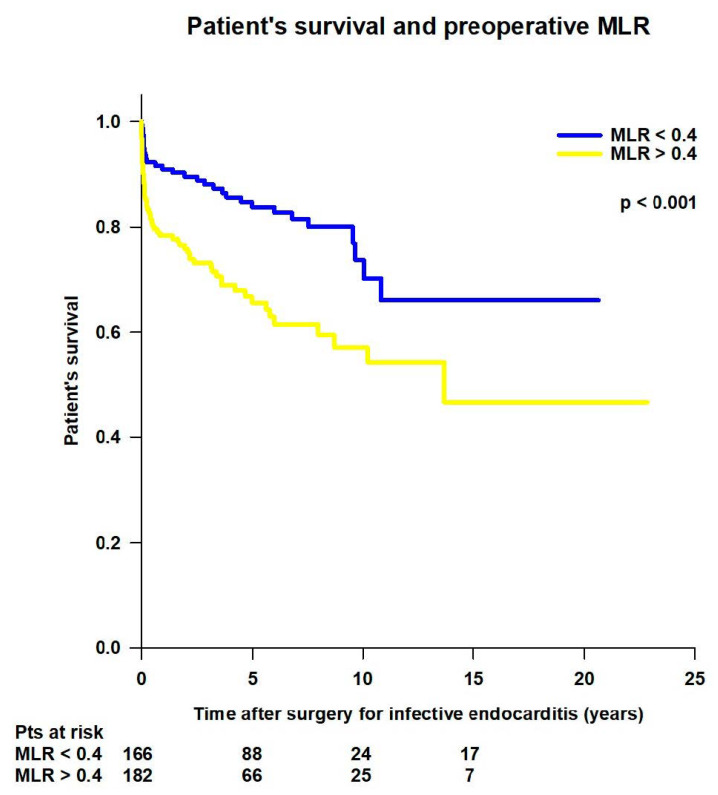
Patient’s survival after surgery for infective endocarditis, according to preoperative monocyte-to-lymphocyte ratio (MLR) value.

Patients with preoperative MLR < 0.4 had significantly better mean survival time (15.6 ± 0.9 vs. 13 ± 1.1 years; *p* < 0.001) compared to patients with preoperative MLR > 0.5 (Figure 9).

### 3.4. Reoperation

Forty (8%) patients underwent reoperation during the follow-up period, 22 (7%) patients with prior NVE and 18 (11%) with prior PVE. Indications for reintervention were recurrent IE (n = 20, 4%), homograft/prosthesis SVD (n = 5, 1%), NSVD (n = 8, 2%), and other (n = 7, 1%).

**Figure 10:**
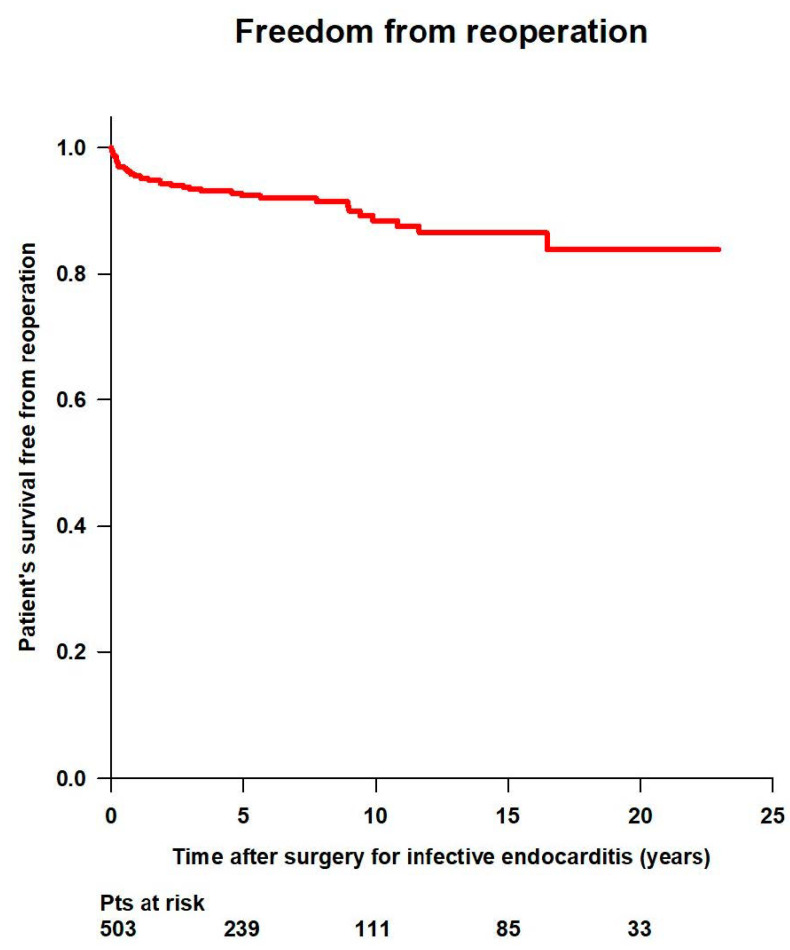
Freedom from reoperation after surgery for infective endocarditis.

Overall survival rates free from reoperation were 99% at 30 days, 95.6% at 1 year, 92.4% at 5 years, 88.4% at 10 years, 86.5% at 15 years, and 83.9% at 20 years (Figure 10).

**Figure 11:**
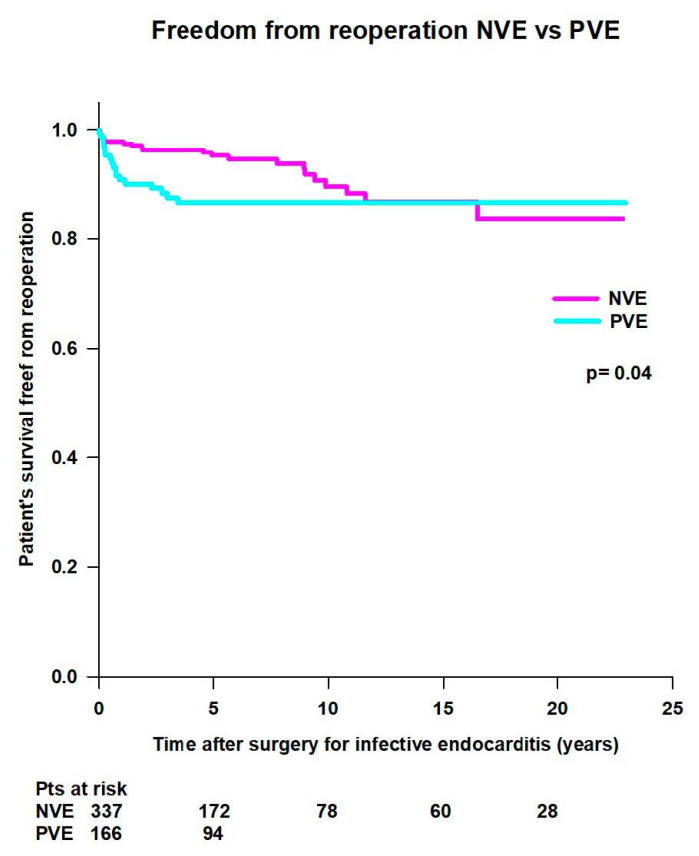
Freedom from reoperation after surgery for native (NVE) and prosthetic (PVE) valve endocarditis.

Mean survival time free from reoperation (20.5 ± 0.5 years vs. 20 ± 0.7 years; *p* = 0.04) and survival rates free from reoperation (99.1% at 30 days, 97.8% at 1 year, 95.2% at 5 years, 89.5% at 10 years, 86.7% at 15 years, and 83.6% at 20 years vs. 93% at 30 days, 90.8% at 1 year, and 86.5% at 5 years) were significantly better in patients with NVE compared to patients with PVE (Figure 11).

**Figure 12:**
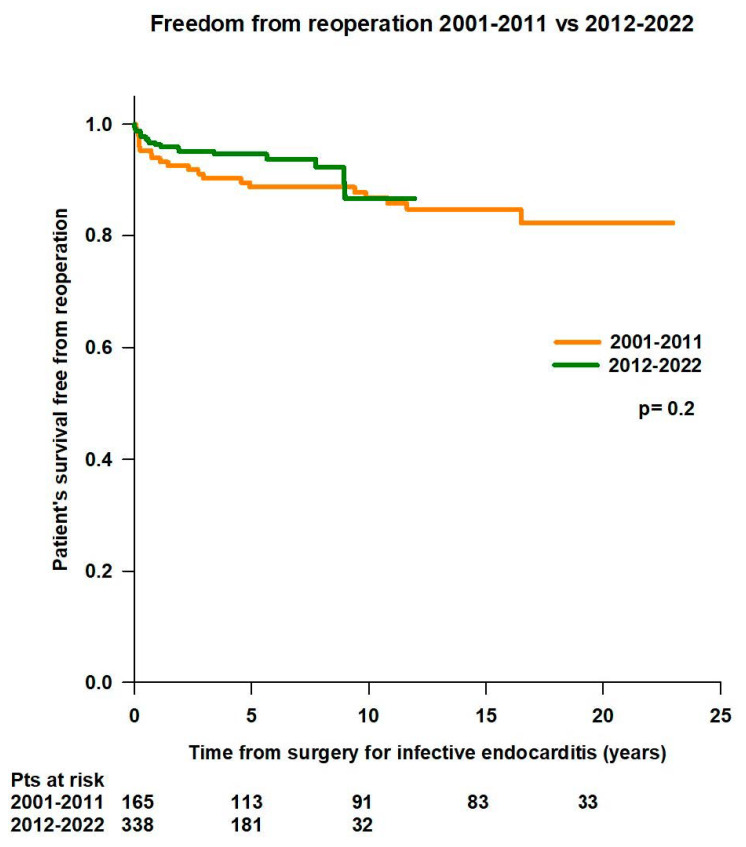
Freedom from reoperation according to the period of surgery for infective endocarditis (2001–2011 vs. 2012–2022).

No difference was observed in in mean survival time (19.9 ± 0.6 years vs. 11.1 ± 0.2 years; *p* = 0.2) and survival rates free from reoperation (94% at 1 year, 88.8% at 5 years, 86.9% at 10 years, 84.8% at 15 years, and 82.3% at 20 years vs. 96.3% at 1 year, 94.7% at 5 years, and 86.7% at 10 years) between patients operated in the first period of the study (2001–2011, n = 165) and patients operated in the second period of the study (2012–2023, n = 338) (Figure 12).

## 4. Discussion

We reported a single-center 20-year experience of surgical treatment of IE and found a remarkable increase of the number of patients who underwent surgery for IE between the first and the second decade of the study. In fact, the number of patients undergoing surgery for IE doubled up, even if the proportion between NVE and PVE remained similar. These findings are consistent with a recent report showing that the incidence of IE has risen in most regions worldwide during the past 30 years [14]. However, despite continuous advances in diagnostic and therapeutic management, we did not find any significant improvement in patients’ survival over time. In our series overall periprocedural mortality was 10%, which is consistent with previous reports showing in-hospital mortality rates ranging from 8% to 24% [15,16,17,18,19,20]. Overall mean survival time was 12.2 ± 0.5 years. However, patients with NVE had better early and late survival compared to patients with PVE, which is in line with prior reports [3,4,5,17]. One of the main findings of our study is that patients who died after surgery or during the follow-up period had significantly higher preoperative values of NLR, NPR, and MLR compared to patients who survived after surgery and during the follow-up period, suggesting that the preoperative inflammatory status may negatively affect short and long-term outcomes. In our series the ROC curve analysis showed that NLR > 3.8 had 62.5% sensitivity and 59.8% specificity in predicting mortality. Additionally, patients with PVE had significantly higher values of NLR, NPR, and MLR compared to patients with NVE, suggesting a more severe disease that may contribute to the worse outcomes observed in these patients. The NLR, PLR, and MLR have been shown to be independent predictors of worse prognosis in many infectious, neoplastic, and cardiovascular diseases [21,22,23,24,25]. Few studies focused on the relationship between hematological parameters and IE. A retrospective study enrolling 121 patients with IE found that high NLR at admission was associated with in-hospital mortality and neurological events and that NLR > 7.1 had an 80% sensitivity and 83% specificity in predicting adverse outcomes [26]. Similarly, another study found that patients with high NLR levels had higher in-hospital mortality, while NLR levels were not predictive of long-term outcomes [27]. Another study analyzed the data from 142 consecutive patients with IE and found that NLR was associated with in-hospital mortality and morbidity and NLR > 8.085 had a 60% sensitivity and an 84.8% specificity for an association with in-hospital mortality [9]. Chen et al. enrolled 678 patients with IE and 2520 healthy controls to evaluate NLR in predicting the early diagnosis and the clinical outcome of IE [10]. The authors showed that the value of NLR was significantly higher in IE patients than healthy controls and that the critical value of NLR for diagnosis of IE was 2.68, with a sensitivity of 69% and a specificity of 88% [10]. In this series, 537 patients had a bad outcome and 141 patients had a good outcome; the authors showed that NLR was significantly higher in bad clinical outcome patients than in good clinical outcome patients and the critical value of NLR to predict the outcome of IE was 5.557, with a sensitivity of 39.0% and a specificity of 85.3% [10]. The role of hematological parameters has also been evaluated in patients with infectious complications related to the presence of cardiac implantable electronic devices (CIED), which may range from local pocket infection (PI) to lead-related infectious endocarditis (LRIE) [28]. The authors found that NL and NPR were significantly higher in patients with LRIE compared to patients with isolated PI [28]. NLR and NPR were characterized by high specificity in the initial diagnosis of CIED infections, with optimal cutoff values of 3.06 and 0.02, compared to CRP; NLR was also shown to be useful in the assessment of the spread of infection in patients with PI, with an optimal cutoff value of 3.13, while NPR may be helpful in the differentiation between vegetation and vegetation-like masses with an optimal cutoff value of 0.03 [28].

Platelets are involved in the pathogenesis of IE, and thrombocytopenia can be observed in 20–25% of patients with IE and is associated with a higher mortality risk in these patients [29,30]. According to previous reports [9,31], in our series PLR had no significant association with in-hospital mortality or in-hospital complications.

Systemic immune-inflammatory index, which integrates three types of inflammatory cells (neutrophil, lymphocytes, and platelets), has been demonstrated to be a promising predictor of adverse events in many immune-related, inflammatory, infectious, neoplastic, and cardiovascular diseases [32,33]. Two studies have found that high SII levels are independently associated with mortality and embolic events in patients with IE [10,34]. However, in our series preoperative levels of SII were not associated with outcome in these patients.

The interest in delineating the diagnostic and prognostic role of biomarkers in IE is constantly evolving. Proteomic analysis studies have identified new potential biomarkers for IE, including NT-proBNP, troponins, cystatin C, matrix metalloproteinase-9, S100 calcium-binding protein A11, lipopolysaccharide-binding protein, VCAM-1, E-selectin, aquaporin-9, CD62E, and IL-6 [6,35]. Future studies are needed to assess the potential diagnostic and prognostic usefulness of these biomarkers. Meanwhile, calculation of the NLR represents an easy and inexpensive method that can be performed at hospital admission and may contribute to early risk stratification of patients with IE.

The study has some limitations. This is a retrospective observational study, and selection bias is unavoidable. The study includes only patients who underwent surgery for infective endocarditis, but we do not know about patients who were too sick for surgery or who were treated conservatively.

The study period includes two decades during which pre and postoperative management of the patients could have changed. Additionally, preoperative hemograms with complete blood counts were not available for all patients.

## 5. Conclusions

Risk stratification is of paramount importance in patients with IE to identify those patients who will take most advantage of surgery. Preoperative higher NLR, NPR, and MLR are associated with increased early and long-term mortality in patients undergoing surgery for IE. Therefore, calculation of the hematological parameters at hospital admission may contribute to the assessment of the optimal timing of surgery and to the improvement of surgical outcomes in patients with IE.

## Figures and Tables

**Table 1:** Aetiology of native and prosthetic valve endocarditis.

Isolated Microorganism	ALL (n = 503)	NVE (n = 337)	PVE (n = 166)	*p*
GRAM+	328 (65%)	216 (64%)	112 (67%)	0.6
* Staphylococcus aureus*	78 (16%)	62 (18%)	16 (10%)	0.01
* Coagulase negative staphylococcus*	75 (15%)	32 (9%)	43 (25%)	<0.001
* Enterococcus* spp.	57 (11%)	35 (10%)	22 (13%)	0.4
* Streptococcus* spp.	99 (20%)	78 (23%)	21 (13%)	0.008
Other GRAM+	19 (4%)	9 (3%)	10 (6%)	0.1
GRAM-	15 (3%)	7 (2%)	8 (5%)	0.1
Fungi	10 (2%)	4 (1%)	6 (4%)	0.1
Negative blood tests	81 (16%)	66 (20%)	15 (9%)	0.004
Multiple species infection	24 (5%)	12 (4%)	12 (7%)	0.1
Unknown	77 (16%)	47 (14%)	30 (19%)	0.1

**Table 2:** Pre, intra and perioperative patients’ characteristics.

	ALL (n = 503)	NVE (n = 337)	PVE (n = 166)	*p*
*Preoperative characteristics*				
Male	371 (74%)	252 (75%)	119 (72%)	0.5
Age, years	65 (52–73)	61 (49–71)	70 (63–75)	<0.001
BMI	25 (23–28)	25 (22–27)	26 (23–29)	0.008
BSA	1.9 (1.7–2)	1.9 (1.7–2)	1.9 (1.7–2)	0.4
Aortic valve IE	352 (70%)	210 (62%)	142 (85%)	<0.001
Mitral valve IE	211 (42%)	171 (51%)	40 (25%)	<0.001
Tricuspid valve IE	26 (5%)	14 (4%)	12 (7%)	0.2
Pulmonary valve IE	4 (1%)	0	4 (2%)	-
Multiple valve IE	84 (17%)	57 (17%)	27 (16%)	0.9
Active IE	330 (66%)	241 (72%)	89 (54%)	<0.001
Cardiogenic/septic shock	45 (9%)	36 (11%)	9 (5%)	0.07
Early IE	46 (9%)	0	46 (28%)	-
Redo surgery	189 (37%)	23 (7%)	166 (100%)	-
*Intraoperative findings*				
Vegetations	305 (61%)	238 (71%)	67 (40%)	< 0.001
Aortic annulus abscess	146 (29%)	51 (15%)	95 (57%)	<0.001
Mitro-aortic discontinuity	48 (10%)	26 (8%)	22 (13%)	0.06
Valve perforation	186 (37%)	166 (49%)	20 (12%)	<0.001
Prosthesis detachment	53 (11%)	0	53 (32%)	-
Gerbode defect	8 (2%)	0	8 (5%)	-
Aortic-left atrium fistula	6 (1%)	1 (0.3%)	5 (3%)	0.02
Interventricular communication	6 (1%)	0	6 (4%)	-
*Surgical technique*				
Aortic valve repair	8 (2%)	8 (2%)	0	-
Aortic valve replacement	284 (56%)	178 (53%)	106 (64%)	0.02
Biological prosthesis	223 (44%)	146 (43%)	77 (46%)	0.5
Mechanical prosthesis	28 (6%)	21 (6%)	7 (4%)	0.4
Aortic homograft Freehand subcoronary technique	33 (7%)	11 (3%)	22 (13%)	<0.001
Aortic root replacement	63 (13%)	26 (8%)	37 (22%)	<0.001
Biological Bentall	15 (3%)	5 (1%)	10 (6%)	0.01
Mechanical Bentall	3 (1%)	1 (0.3%)	2 (1%)	0.5
Aortic homograft Full root technique	28 (6%)	7 (2%)	21 (13%)	<0.001
Aortic homograft Intraluminal cylinder technique	14 (3%)	10 (3%)	4 (2%)	0.9
Ross procedure	3 (1%)	3 (1%)	0	-
Mitral valve repair	66 (13%)	55 (16%)	11 (7%)	0.004
Mitral valve replacement	166 (33%)	130 (39%)	36 (22%)	<0.001
Biological prosthesis	136 (27%)	102 (30%)	34 (20%)	0.02
Mechanical prosthesis	30 (6%)	28 (8%)	2 (1%)	0.003
Tricuspid valve repair	16 (3%)	10 (3%)	6 (4%)	0.9
Tricuspid valve replacement	7 (1%)	6 (2%)	1 (1%)	0.5
Biological prosthesis	6 (1%)	6 (2%)	0	-
Pulmonary homograft	1 (0.2%)	0	1 (1%)	-
Pulmonary valve replacement	4 (1%)	0	4 (2%)	-
Biological prosthesis	1 (0.2%)	0	1 (1%)	-
Pulmonary homograft	3 (1%)	0	3 (2%)	-
Ascending aorta replacement	15 (3%)	7 (2%)	8 (5%)	0.1
Coronary artery-bypass grafting	35 (7%)	27 (8%)	8 (5%)	0.2
Aortic Homograft	75 (15%)	28 (8%)	47 (28%)	<0.001
CPB time, min	123 (91–180)	105 (84–145)	180 (131–235)	<0.001
Cross-clamping time, min	95 (69–134)	82 (64–114)	129 (96–175)	<0.001
Intra-aortic ballon pump	32 (6%)	22 (7%)	10 (6%)	0.9
ECMO	14 (3%)	8 (2%)	6 (4%)	0.6
Intraoperative mortality	6 (1%)	2 (1%)	4 (2%)	0.1
*Perioperative characteristics*				
Re-exploration for bleeding	38 (8%)	22 (7%)	16 (10%)	0.2
Pacemaker implantation	45 (9%)	20 (6%)	25 (15%)	<0.001
Cerebrovascular accident	12 (2%)	6 (2%)	6 (4%)	0.3
Continuous renal replacement therapy	25 (5%)	14 (4%)	11 (7%)	0.3

**Table 3:** Preoperative median values of neutrophil-to-lymphocyte ratio (NLR), neutrophil-to-platelet ratio (NPR), platelet-to-lymphocyte ratio (PLR), monocyte-to-lymphocyte ratio (MLR), neutrophil-to monocyte ratio (NMR), and systemic inflammatory index (SII) in the whole cohort, in patients with native (NVE) and prosthetic valve endocarditis (PVE), and in survivors and non-survivors after surgery for infective endocarditis.

	ALL (n = 317)	NVE (n = 224)	PVE (n = 93)	*p*	Survivors (n = 229)	Non-Survivors (n = 88)	*p*
NLR	3.7 (2.4–6.1)	3.3 (2.3–6)	4.2 (2.8–6.6)	0.05	3.3 (2.3–5.5)	4.6 (2.9–7.6)	< 0.001
NPR	23 (16–37)	21 (16–36)	29 (20–41)	0.007	22 (15–34)	29 (18–49)	< 0.001
PLR	159 (106–210)	160 (108–213)	157 (104–201)	0.6	160 (110–208)	158 (101–234)	0.9
NMR	8.4 (6.6–12)	8.5 (6.6–12.3)	8 (6.2–11.4)	0.2	8.3 (6.5–11.9)	8.5 (6.7–13.2)	0.5
MLR	0.41 (0.29–0.62)	0.39 (0.28–0.58)	0.47 (0.35–0.74)	0.001	0.39 (0.28–0.58)	0.55 (0.33–0.75)	< 0.001
SII	790 (485–1396)	732 (456–1363)	875 (524–1417)	0.2	745 (466–1305)	863 (561–1645)	0.1

**Table 4:** Predictors of mortality at univariate at multivariate analysis.

	Univariate Analysis		Multivariate Analysis	
	Hazard Ratio (95%CI)	*p*	Hazard Ratio (95%CI)	*p*
Age > 65 years	2.25 (1.69–3.01)	<0.001	2.07 (1.53–2.82)	<0.001
Male sex	0.81 (0.6–1.1)	0.18		
BMI > 25	1.43 (1.09–1.87)	0.01	1.31 (0.99–1.73)	0.05
PVE	1.45 (1.09–1.92)	0.009	1.06 (0.79–1.44)	0.36
Postoperative IABP	3.21 (2.03–5.09)	<0.001	1.73 (0.93–3.19)	0.07
Postoperative ECMO	6.47 (3.49–11.9)	<0.001	2.86 (1.29–6.36)	0.01
Postoperative CVA	1.29 (0.72–2.29)	0.38		
Postoperative CRRT	5.27 (3.3–8.43)	<0.001	3.36 (2.02–5.62)	<0.001

BMI: body mass index; CRRT: continuous renal replacement therapy; CVA: cerebrovascular accident; ECMO: extracorporeal membrane oxygenation; IABP: intra-aortic balloon pump; PVE: prosthetic valve endocarditis.

## Data Availability

The data presented in this study are available upon request from the corresponding author.
